# Copy number gain of *MYCN* gene is a recurrent genetic aberration and favorable prognostic factor in Chinese pediatric neuroblastoma patients

**DOI:** 10.1186/1746-1596-8-5

**Published:** 2013-01-15

**Authors:** Miao Wang, Chunju Zhou, Rongqin Cai, Yong Li, Liping Gong

**Affiliations:** 1 Department of Pathology, Basic Medical College, Capital Medical University, Beijing, China; 2 Department of Pathology, Beijing Children’s Hospital, Capital Medical University, Beijing, China

## Abstract

**Background:**

Amplification of *MYCN* oncogene is an established marker indicating aggressive tumor progression of neuroblastoma (NBL). But copy number analyses of *MYCN* gene in ganglioneuroblastoma (GNBL) and ganglioneuroma(GN) is poorly described in the literature. In the study, we evaluated the copy number aberrations of *MYCN* gene in clinical samples of NBLs, GNBLs and GNs and analyzed their association with clinical outcome of the patients.

**Methods:**

In this study, we analyzed *MYCN* gene and chromosome 2 aneusomy by using fluorescence in situ hybridization (FISH) method in a total of 220 patients with NBL, GNBL and GN cases. Kaplan-Meier curves were generated by using SPSS 12.0 software.

**Results:**

Of 220 patients, 178 (81.0%) were NBLs, 32 (14.5%) were GNBLs and 10 (4.5%) were GNs. *MYCN* gain is a recurrent genetic aberration of neuroblastic tumors (71.8%, 158/220), which was found in 129 NBLs (58.6%, 129/220), 25 GNBLs (11.4%, 25/220) and 4 GN cases (1.8%, 4/220). However, *MYCN* amplification was only present in 24 NBL tumors (13.5%, 24/178) and 1 GNBL case (3.1%, 1/32). Kaplan-Meier survival analysis indicated that *MYCN* amplification is significantly correlated with decreased overall survival in NBLs (P=0.017). Furthermore, a better prognosis trend was observed in patients with *MYCN* gain tumors compared with those with *MYCN* gene normal copy number tumors and *MYCN* amplification tumors (P=0.012).

**Conclusions:**

In summary, the frequency of *MYCN* amplification in NBLs is high and is rarely observed in GNBLs and GNs, which suggest *MYCN* plays an important role in neuroblastic tumors differentiation. *MYCN* gain appeared to define a subgroup of NBLs with much better outcome and classification of *MYCN* gene copy number alteration as three groups (amplification, gain and normal) can provide a powerful prognostic indicator in NBLs.

**Virtual Slides:**

The virtual slide(s) for this article can be found here: http://www.diagnosticpathology.diagnomx.eu/vs/6417541528559124

## Background

Peripheral neuroblastic tumors (NTs) including neuroblastoma(NBL), ganglioneuroblastoma(GNBL), and ganglioneuroma(GN) comprise one of the most common groups of neoplastic disease in infants and children. NBL and GNBL are considered malignant. In contrast, GNs are considered as benign tumors. In histology, NBL, GNBL, and GN can be conceptualized as three maturational manifestations of a common neoplasm [1].

Amplification of *MYCN* oncogene is an established marker indicating aggressive tumor progression of NBL [2,3]. Brodeur et al. [4] were the first to show that *MYCN* amplification occurs in a substantial subset of primary untreated NBLs and is highly correlated with advanced stage. Seeger et al. [5,6] then demonstrated a strong association with rapid disease progression and a poor prognosis. Analysis of *MYCN* remains an essential component of disease evaluation for newly diagnosed NBL patients and serves as a paradigm for the utility of molecular biologic information in cancer treatment stratification [7-9]. *MYCN* is vital for proliferation, migration and stem cell homeostasis while decreased levels are associated with terminal neuronal differentiation [10]. On the other hand, downregulation of *MYCN* leads to decreased proliferation and differentiation, emphasizing the importance of MYC signaling in NBL biology [11,12]. But copy number status of *MYCN* gene in GNBL and GN is poorly described in the literature [13].

In the study, we evaluated the copy number aberrations of *MYCN* gene in formalin-fixed, paraffin-embedded clinical samples of NBLs, GNBLs and GNs and analyzed their association with clinical outcome of the patients.

## Methods

### Tumor tissue and patient information

Formalin-fixed, paraffin-embedded clinical samples taken from 220 pediatric neuroblastic tumors enrolled on therapeutic or nontherapeutic protocols between 2009 and 2011. Specimen was limited to patients whose diagnosis of neuroblastic tumors was based on histologic and immunohistochemistry examination. Selected clinical and laboratory data (e.g., age at diagnosis, sex, tumor site) were retrieved from the Beijing Children Hospital. The patient characteristics are described in Table 1.

**Table 1:** Patient characteristics

**Characteristics**	**GN**	**GNBL**	**NBL**
**Patients [no. % ]**	10	32	178
**Median age (years)**	3	3.5	1.8
**Male/female ratio**	4/6	14/18	113/65
**Sites**			
**adrenals**	1	12	67
**thorax**	6	12	31
**abdomen**	2	8	50
**pelvis**	0	0	10
**others**	1	0	20
***MYCN *status**			
**normal**	6	6	25
**gain**	4	25	129
**amplification**	0	1	24

GN :Ganglioneuroma; GNBL: Ganglioneuroblastoma; NBL: Neuroblastoma.

### Fluorescence in situ hybridization (FISH)

*MYCN* gene was investigated by interphase FISH on paraffin sections as previously described [14]. Briefly, the 4 μm-thick tissue sections were deparaffinized and pressure-cooked in 1mM ethylene diamine tetraacetic acid (EDTA) buffer for 3 min. The tissues were then digested in 0.1% pepsin solution at 37°C for 20 min, dehydrated and added with the appropriate probes. *MYCN SG/CEP2 SO* probe is used in this study (Vysis, Abbott Laboratories, Abbott Park, IL). The slides were incubated at 80°C for 25 min and at 45°C for 2 days. The slides were then washed in post-hybridization buffers, stained with anti-fade solution containing 4’ ,6-diamidino-2-phenylindole (DAPI; Vector Labs, Burlingame, CA) and examined using a fluorescence microscope (BX51; Olympus, Tokyo, Japan) by two investigators independently. Slides with known structural or numerical abnormality for the above probes were used as positive controls, and a case of reactive hyperplasia of the tonsil was used as a negative control.

### FISH scoring scheme

Fluorescence microscopy was performed with a BX51 microscope equipped with filter set for FITC, Texas red, and DAPI. Each sample was analyzed to determine the origin of the amplification unit (extrachromosomal double minutes or intrachromosomal homogeneously staining regions) and the proportion of cells with amplified *MYCN* genes. The FISH signals were scored in 200 no overlapping nuclei per core, independently by two investigators (M Wang and LP Gong.), and the consensus was recorded. Four cellular groups were defined as previous study [15]. No Alteration: cells with 2 *MYCN* signals and 2 CEP2 signals; Amplification: The number of *MYCN* signals is at least 10 copies greater than the control probe signals;Loss/Imbalance:Presence of at least 2 *MYCN* signals and increased CEP2 signals;Gain: The number of MYCN signals is 1–9 copies more than CEP2 signals.

### Statistical analysis

SPSS 12.0 software (SPSS, Chicago, IL) was used to analyze differences in neuroblastic tumor characteristics among the patient groups. The χ2 test or two-tailed Fisher’s exact test was used to compare the predictive values of each marker analyzed. A p value of less than 0.05 was considered statistically significant. Kaplan-Meier curves were generated by using SPSS 12.0 software.

## Results

### Clinical features

We analyzed a panel of 220 pediatric patient samples comprising 178 NBLs, 32 GNBLs, and 10 GNs. Patient characteristics are summarized in Table 1. There was a male predominance in NBL disease group, with the male to female ratio being 1.7:1. The median age at diagnosis was 3 years for GNs, 3.5 years for GNBLs and 1.8 years for NBLs. Patients with NBL were significantly younger than patients with GN and GNBL (P < 0.001). The distribution of these three diseases generally follows the distribution of the sympathetic ganglia. In our study, about 36.4% (80/220) tumors arise in adrenal gland. In addition, adrenal involvement at diagnosis was 1 case for GN, 12 cases for GNBL and 67 cases for NBL.

### Morphology features

**Figure 1:**
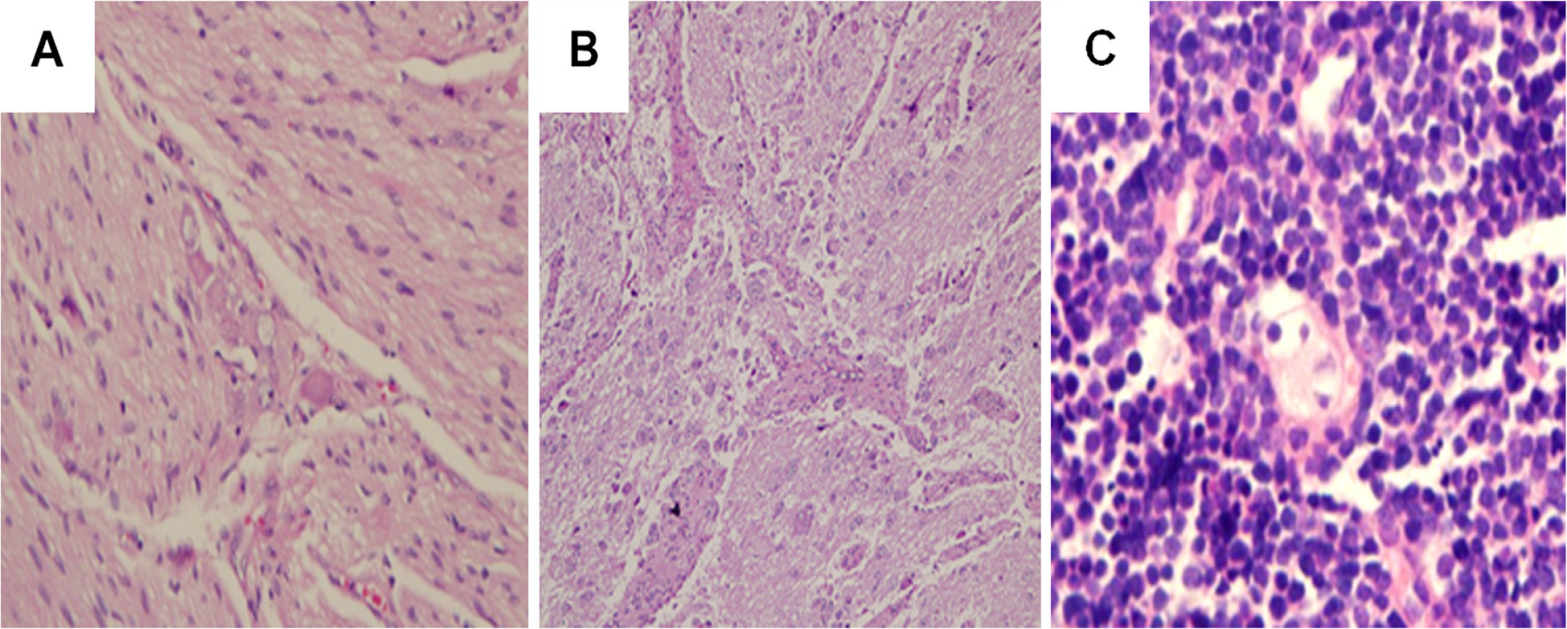
**Representative HE image of neuroblastic tumors. A**, Ganglioneuroma: Mature ganglion cell with schwannian stroma. **B**, Ganglioneuroblastoma: Increased schwannian stroma. **C**, Neuroblastoma: A monotonous population of hyperchromatic cells with scant cytoplasm.

Neuroblastic tumors were divided into three histological subtypes on morphologic criteria of neuroblastic tumors which was recommendations by the International Neuroblastoma Pathology Committee [16]. GN, a fully differentiated tumor, is characterized by a mixture of mature schwann cells and ganglion cells (Figure 1A). GNBL has primitive neuroblasts along with maturing ganglion cells (Figure 1B); the number and arrangement of the cells vary so the tumor assumes a wide range of appearances. NBL, the least differentiated, resembles the fetal adrenal medulla and is composed of primitive neuroblasts (Figure 1C). GNBLs and GNs are usually of favorable histology. In fact, GN is considered a benign neuroblastic tumor.

### *MYCN* gene status analysis

*MYCN* gene FISH analysis was successful in all cases of GN, GNBL and NBL, and the results are detailed in Table 1. *MYCN* status was determined in light of chromosome 2 copy number. No cases showed loss of centromere 2 and *MYCN*. But in total, 158 cases with *MYCN* gene gain also showed gain of centromere 2, suggesting polyploidy in these cases.

**Figure 2:**
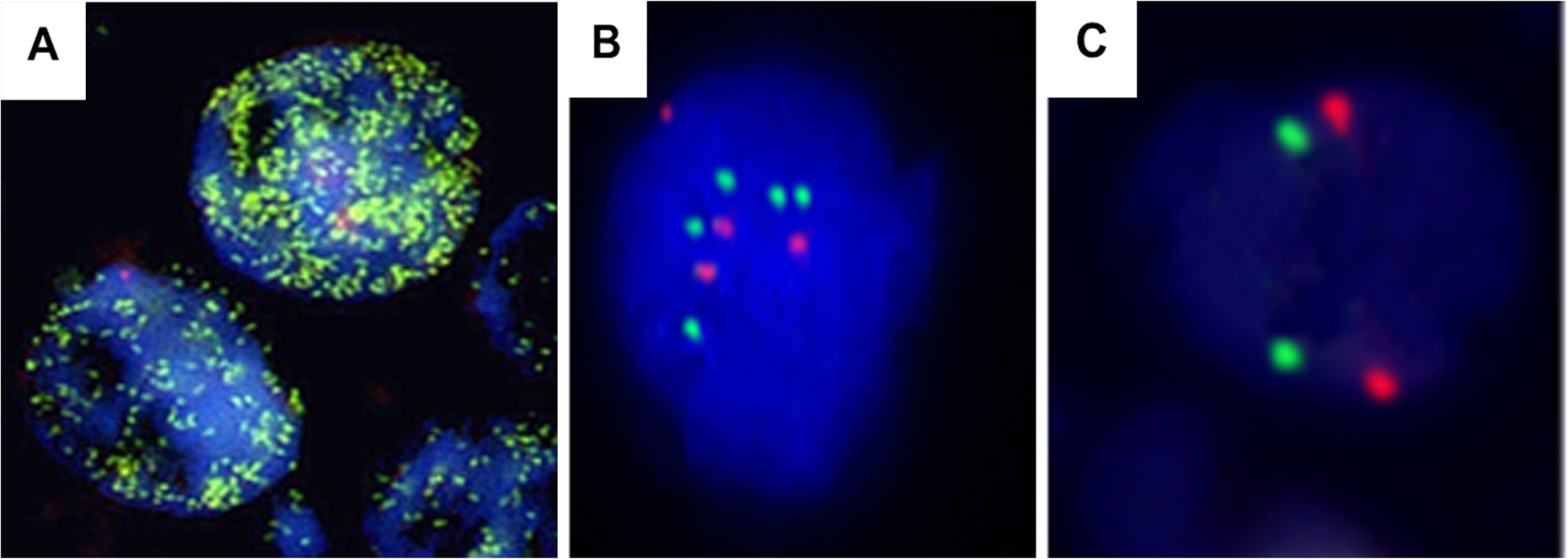
**Representative FISH image of neuroblastic tumor cells displaying *MYCN *gene status. A**: Amplification:The number of *MYCN* signals (green) is more than 10 copies of the *CEP2* probe signals (red). **B**: Gain:The number of *MYCN* signals (green) is 1 copy greater than the *CEP2* probe signals; **C**: No Alteration: Cells with *MYCN* signals (green) showing the same numbers of the *CEP2* probe signals. (DAPI counterstain, original magnification ×1000).

Among 178 NBL cases, 153 (85.9%) were *MYCN* gene alterations, including 24 cases (13.5%) with *MYCN* amplification [Figure 2A] and 129 cases (72.5%) with *MYCN* gain [Figure 2B]. *MYCN* gene normal [Figure 2C] was only found in 25 cases (14.0%) of NBL, which was significantly lower than in GNBL and in GN (p<0.05).

In GN and GNBL cases, six of ten (40.0%) and six of thirty-two (18.8%) cases showed no aberration of *MYCN* gene, respectively. The frequency of *MYCN* gain was significantly higher in GNBL (78.1%, 25/32) than in GN (40.0%, 4/10) (p<0.05).

### Prognostic analysis

**Figure 3:**
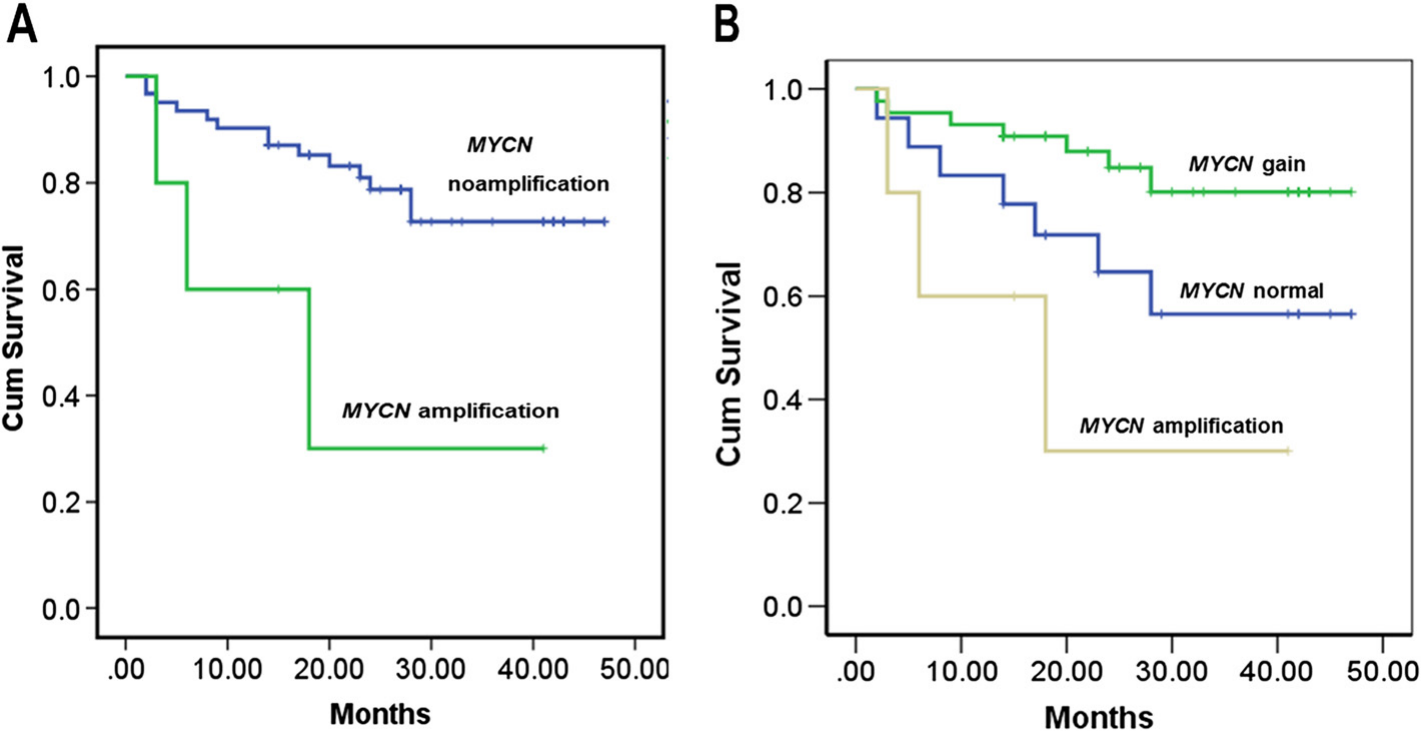
**Overall survival analysis. A**. *MYCN* amplification is correlated with decreased overall survival in NBL. P=0.017. **B**. *MYCN* amplification is correlated with decreased overall survival and *MYCN* gene gain is correlated with good outcome in NBL. P=0.012.

Sixty-seven NBL cases had follow-up information available, with 50 survived and 17 died at the time of writing up of the study. The patients typically received multiagent chemotherapy. The survival time ranged from 0 to 47 months and the average survival time was 37.4 months. A significant trend was observed between *MYCN* gene amplification tumors and poor outcome compared with those with no amplification of *MYCN* gene patients (p=0.017) (Figure 3A). Interestingly,the Kaplan-Meier survival analysis also indicated a significant better prognosis in patients with *MYCN* gene gain tumors compared with those with *MYCN* gene normal tumors (p=0.012) (Figure 3B).

## Discussion

The peripheral neuroblastic tumour group includes NBL, GNBL and GN. NBL is the most common extracranial solid tumour of childhood and the incidence of pediatric neuroblastoma are increasing [17,18]. *MYCN* gene amplification is a known molecular marker for aggressive progression of NBL [4]. In the study, we evaluated the histological presentation and *MYCN* gene copy number in 220 pediatric neuroblastic tumors, which include 178 NBLs, 32 GNBLs and 10 GNs and analyzed their association with clinical outcome of the patients. To our knowledge, this is the first article for *MYCN* gene and chromosome 2 aneusomy analyses by using fluorescence in situ hybridization (FISH) method in chinese pediatric patients.

Our study reaffirmed the need for *MYCN* copy number to be determined in light of chromosome 2 copy number. *MYCN* copy number had been determined by southern blot analysis [19]. After 1993, fluorescence in situ hybridization (FISH) was used to determine the presence of *MYCN* amplification [20,21]. In these studies, the results of southern blotting and FISH analysis were prospectively compared and a *MYCN* copy number of ≥ 10 was determined to be the optimal cutoff by FISH [20], as the vast majority of amplified tumors have very large numbers of double minutes in each tumor cell. By southern blotting, any normal cells in the tissue were included in the measurement, whereas by FISH, each tumor nucleus was visualized directly and simultaneous cohybridization with a specific chromosome probe is of great value in predicting the prognosis of patients [22]. FISH has a higher sensitivity because it detects the *MYCN* copy number on the single-cell level and allows correlation of morphologic details. In our estimation, FISH is a practical, useful and reliable method for analysis of *MYCN* copy number in neuroblastic tumors.

Our results showed that aberrant *MYCN* copy number was detected in 153 (85.9%) of 178 NBLs, with amplification constituting 24 (13.5%), gain 129(72.4%). In contrast, *MYCN* amplification is only observed in one GNBL case (1/32, 3.1%) and no GN cases (0/10, 0%). Moll A et al. [23] also reported that no amplification of the *MYCN*-oncogene was found in mixed hepatoblastoma and teratoma of the liver in a 3-year-old boy. Wan,T.S et al. [24] investigated 12 NBL patients for *MYCN* amplification by FISH and found that 16.7% cases had *MYCN* amplification. Angelini,P et al. [13] reported that only about 2% had *MYCN* gene amplified tumours in 232 GNBL patients. Our results also showed that the frequency of MYCN gene gain was significantly higher in GNBL (78.1%, 25/32) and NBL (72.5%, 129/178) than in GN (40.0%, 4/10). Toraman,A.D et al. [25] found that chromosomal gains displayed by chromosomes and chromosome loci were 2p25 approximately pter (60%) in five GNBL cases by comparative genomic hybridization. Truong LN et al. [26] also detected *MYCN* oncogenes in malignant brain tumors by using multiplex ligation dependent probe amplification (MLPA). Thus, higher frequency of *MYCN* gene aberrations in undifferentiated or less differentiated tumors indicates an important function of *MYCN* gene in tumor malignancy.

*MYCN* amplification is an established marker indicating aggressive tumor progression of NBL [27]. Our data also showed that *MYCN* amplification is correlated with decreased overall survival in NBL (P=0.017) (Figure 3A). More significantly, we demonstrated for the first time that the presence of extra copies of *MYCN* gene is an independent prognostic factor for NBL in our case series. The patients with *MYCN* gene gain had a significantly longer mean survival time than those with normal *MYCN* gene copy number (P=0.012) (Figure 3B). In our data, NBL cases with *MYCN* gene gain also showed gain of centromere 2, suggesting polyploidy in these cases. In 1991, look et al. [28] found that NBL patietns treated with cyclophosphamide-doxorubicin, hyperdiploidy was closely associated with long-term disease-free survival (greater than 90% of cases), while diploidy invariably predicted early treatment failure (P <0.001). Recently, George et al. had also found that NB patients with hyperdiploidy plus no amplified *MYCN* confers a favorable prognosis [29], which is in line with our study. Furthermore, they also found that hyperdiploidy plus no amplified *MYCN* NBL patients may respond well to contemporary chemotherapy, and could be spared intensive myeloablative therapy with stem-cell rescue [29]. Thus, the classification of *MYCN* gene status as three groups by FISH may provide more powerful prognostic indicator and better treatment options in NBL.

## Conclusions

In summary, using simple and easily applicable FISH technique we showed in the present study that the frequency of *MYCN* amplification in NBLs is high and is rarely observed in GNBLs and GNs, which suggest *MYCN* gene play an important role in neuroblastic tumors differentiation. Furthermore, the copy number gain of *MYCN* gene locus appeared to define a subgroup of NBL with much better outcome and classification of *MYCN* gene copy number alteration as three groups (amplification, gain and normal) can provide a powerful prognostic indicator in NBL.

## Abbreviations

GN: Ganglioneuroma; GNBL: Ganglioneuroblastoma; NBL: Neuroblastoma; FISH: Fluorescence in situ hybridization.

## Competing interests

The authors declare that they have no competing interests.

## Authors’ contributions

Miao Wang, Yong Li and Rongqin Cai carried out the FISH studies. Miao Wang drafted the manuscript. Miao Wang, Chunju Zhou and Liping Gong participated in the design of the study. Chunju Zhou participated in and coordinated specimen and clinical data retrieval and characterization. Liping Gong conceived and coordinated the study. All authors read and approved the final manuscript. This study was conducted with approval from Capital Medical University.
